# Preferences for mobile HIV pre-exposure prophylaxis delivery among young men with alcohol use in rural South Africa: A discrete choice experiment

**DOI:** 10.4102/sajhivmed.v27i1.1840

**Published:** 2026-10-07

**Authors:** Alison C. Castle, Nkosinathi I. Mthembu, Lucky Nzimande, Thenjiwe Ncube, Bongani Nkosi, Sweetness Dube, Jana Jarolimova, Susanne S. Hoeppner, Maryam Shahmanesh, Mark J. Siedner

**Affiliations:** 1 Division of Infectious Diseases, Massachusetts General Hospital, Boston, United States of America; 2 Africa Health Research Institute, Durban, South Africa; 3 Department of Medicine, Harvard Medical School, Boston, United States of America; 4 Division of Infectious Diseases, Brown University Health, Providence, United States of America; 5 Department of Medicine, Brown University Health, Providence, United States of America; 6 Department of Psychiatry, Massachusetts General Hospital, Boston, United States of America; 7 Department of Infection and Population Health, University College London, London, United Kingdom; 8 School of Clinical Medicine, College of Health Sciences, University of KwaZulu-Natal, Durban, South Africa

**Keywords:** HIV prevention, pre-exposure prophylaxis, men, alcohol use, delivery preferences

## Abstract

**Background:**

Young men who use alcohol have an elevated risk for HIV in South Africa but have low engagement with pre-exposure prophylaxis (PrEP).

**Objectives:**

We conducted a discrete choice experiment (DCE) to explore preferences for mobile HIV prevention services in rural KwaZulu-Natal.

**Method:**

Men aged ≥ 18 years with self-reported alcohol use participating in an HIV-prevention clinical trial completed a DCE. Attributes included clinic location (soccer fields, shebeens [informal bars], taxi ranks), operating hours (weekday, week-evening, weekend), and HIV prevention modality (event-driven oral PrEP, injectable PrEP, condoms only). Preferences were estimated using hierarchical Bayesian multinomial logit models. Relative attribute importance quantified each attribute’s contribution to decision-making. Exploratory subgroup analyses examined variation by prior PrEP use, alcohol risk (AUDIT-C > 4), and age.

**Results:**

Among 185 participants (mean age 27 years), HIV prevention modality was the most influential attribute (62.8% relative importance), followed by clinic location (26.3%) and clinic timing (10.9%). Services located at soccer fields were strongly preferred, while delivery at shebeens was disfavoured. Clinic timing showed minimal variation in preferences. Event-driven oral PrEP was preferred over condoms-only delivery, while injectable PrEP showed mixed preferences. Men with prior PrEP experience and high-risk alcohol use preferred injectable PrEP, whereas PrEP-naïve men and those with lower-risk drinking patterns preferred event-driven oral PrEP.

**Conclusion:**

PrEP programmes for young men should pair prevention choice with acceptable service locations. Event-driven oral PrEP may be an entry point for PrEP-naïve men; injectable PrEP may appeal more to men with prior PrEP experience or higher-risk alcohol use.

**What this study adds:** This study shows that PrEP choice matters for young men as programmes expand differentiated and long-acting options. Preferences varied by prior PrEP experience and alcohol use patterns. Findings also highlight the importance of socially acceptable delivery locations: soccer fields were preferred, while shebeens were disfavoured.

## Introduction

South Africa remains at the epicentre of the HIV epidemic, with high incidence despite free treatment and prevention services. In 2024, the Joint United Nations Programme on HIV/AIDS estimated HIV incidence at 5.3 per 1000 adults aged 15–49.^1^ Although HIV incidence is higher among young women, transmission dynamics point to a critical prevention role for men. In KwaZulu-Natal, phylogenetic evaluation of community-wide transmission networks link many infections in young women aged 15–25 years to older male partners primarily aged 25–40 years, placing adult men at the centre of heterosexual transmission pathways.^2^ This transmission pathway persists because of gender gaps in the HIV care cascade, where men are less likely than women to know their status and achieve viral suppression, ultimately undermining the population-level benefits of treatment-as-prevention.^3^ Consequently, prevention strategies that focus primarily on adolescent girls and young women remain necessary but insufficient.^4^

Oral pre-exposure prophylaxis (PrEP) is an effective biomedical prevention strategy that has been integrated into South Africa’s national HIV prevention programme since 2016; yet PrEP uptake among adolescent boys and young men remains limited. In a population-based prospective study in KwaZulu-Natal, only 13% of PrEP-eligible male patients aged 15–24 years initiated PrEP.^5^ Initiation differed by service entry point, with initiation rates of 4% through facility-based clinics, 18% through mobile clinics, 21% through schools, and 24% through community events.^5^ These differences suggest a delivery mismatch, in which PrEP engagement among men varies depending on how and where services are offered. For example, a mobile clinic programme providing sexual health services in bars (shebeens) in rural KwaZulu-Natal demonstrated that offering HIV testing and daily oral PrEP to shebeen patrons was operationally feasible and acceptable to both men and venue owners.^6,7^ However, despite successfully reaching men at elevated risk for HIV, uptake remained modest, with fewer than one-third of eligible men completing clinical evaluation and initiating PrEP.^7^ This gap between reach and initiation suggests that offering services in high-risk venues may be insufficient, and that other features of service design may influence men’s willingness to engage. However, there is limited evidence on how men prioritise or trade off specific attributes of prevention services, including service location, timing, and prevention modality, when deciding whether to engage.

Alcohol-serving venues may be high-yield points for HIV-prevention delivery because they concentrate both high-risk sexual behaviours and dense male social networks. These settings often lead to multiple sexual partners, sex after drinking, and inconsistent condom use, making the need and potential impact of PrEP greater.^8,9^ However, alcohol-serving venues may also present challenges for health service delivery. Patrons may be focused on social activities or intoxicated, and the social context of these venues may shape men’s willingness to engage with preventive health services. Consistent with this complexity, our work in rural KwaZulu-Natal found that men with hazardous alcohol use were more likely to initiate PrEP (40%) than those with low-risk drinking behaviours (17%) when services were offered through community-based mobile clinics in open community spaces.^10^ However, persistence remained low, with only 27% continuing PrEP 4 months later.^10^ Together, these findings suggest that maximising the preventive impact of PrEP for young men with high-risk behaviours will require delivery models aligned with their preferences for where, how, and when services are accessed.

To address this gap, we conducted a discrete choice experiment (DCE) to quantify young men’s preferences across key attributes of PrEP delivery in rural KwaZulu-Natal, South Africa. These findings may inform implementation strategies as long-acting PrEP formulations, including lenacapavir, are introduced into South Africa’s public-sector prevention programmes.

## Research methods and design

### Study design and setting

We conducted a DCE among young men with alcohol use in rural KwaZulu-Natal, South Africa. The DCE was structured to elicit preferences and trade-offs across predefined attributes of mobile HIV prevention services.^11,12^

Participants were recruited from the *Isisekelo Sempilo* Study, an open-label 2×2 randomised factorial trial conducted in the uMkhanyakude District of KwaZulu-Natal, South Africa, between March 2020 and August 2022.^13^ Briefly, trial participants were recruited from the Africa Health Research Institute’s longitudinal Health and Demographic Surveillance System through random household sampling of individuals aged 16 years to 29 years.^14^ During the 12-month trial, participants received appointments to attend youth-friendly mobile clinics in open fields, where they were offered HIV testing, sexually transmitted infection screening, counselling, and information about PrEP. Those who tested negative for HIV and reported being sexually active were offered free oral daily PrEP (tenofovir disoproxil fumarate and emtricitabine). Participants attended monthly visits during the initiation stage, and thereafter received 3-monthly refills, testing, and counselling.

### Participant selection and recruitment

Men were eligible for the DCE if they were aged ≥ 18 years; had participated in the parent trial; reported any alcohol use during the trial period (AUDIT-C score 1–12)^15^; were living without HIV; and were sexually active at the time of the parent trial. Of the 365 men participating in the parent trial, 240 (66%) met eligibility criteria for the DCE. Participants were identified from the parent trial registry and approached by trained, bilingual research staff at their homes to arrange a convenient time and location for completing the DCE between 04 June 2025 and 14 January 2026. Surveys were administered in person in the local language (isiZulu) using tablet-based data collection, with visual aids to support comprehension. Research staff provided instructions and guided participants through the first-choice task to ensure comprehension without influencing responses; participants completed the remaining choice tasks independently.

### Attribute development

Attributes and attribute-levels were informed by formative qualitative research conducted with the same source population and supplemented by review of the South African PrEP delivery literature. Thirty in-depth interviews were conducted with men purposively sampled across distinct PrEP engagement trajectories following the parent trial (declined PrEP, initiated but discontinued, and continued PrEP).^16^ The semi-structured interview guide included a section on ideal healthcare service models, where participants described preferred features of mobile health services and responded to preliminary service concepts developed by the study team. Interview transcripts were thematically analysed to identify service delivery features that influenced men’s willingness to initiate or continue PrEP.^16^ These findings were then discussed within the study team to develop candidate attributes and levels. Attributes were prioritised if they were repeatedly raised by participants, modifiable within a mobile clinic intervention, and actionable within the South African public-sector context.

The final set of attributes focused on key service delivery features relevant to PrEP uptake and persistence. Daily oral PrEP was excluded as no interview participants expressed interest in this option. Instead, prevention method levels included event-driven (2–1–1) oral PrEP, injectable PrEP, and condoms-only delivery. Injectable PrEP was included to reflect anticipated public-sector rollout of long-acting formulations in South Africa, including the recently approved 6-month injectable lenacapavir. Location attributes were refined to reflect male-dominated social spaces commonly described in interviews, such as soccer fields and alcohol-serving venues (e.g. shebeens), replacing mobile clinic locations in fields and open spaces used in the parent trial. Certain preferences identified in interviews, such as home delivery of PrEP or providing alcohol as an incentive, were excluded because of logistical or ethical constraints. The resulting attributes included clinic location (soccer fields, shebeens, taxi ranks), clinic timing (weekdays, week evenings, weekends), and HIV prevention method (event-driven oral PrEP 2–1–1, injectable PrEP, condoms only) (Figure 1). The final instrument was pilot tested with three male staff members aged 26–38 years to assess clarity and overall completion time. All were from KwaZulu-Natal and spoke isiZulu as their first language; one was from the study community.

**FIGURE 1:**
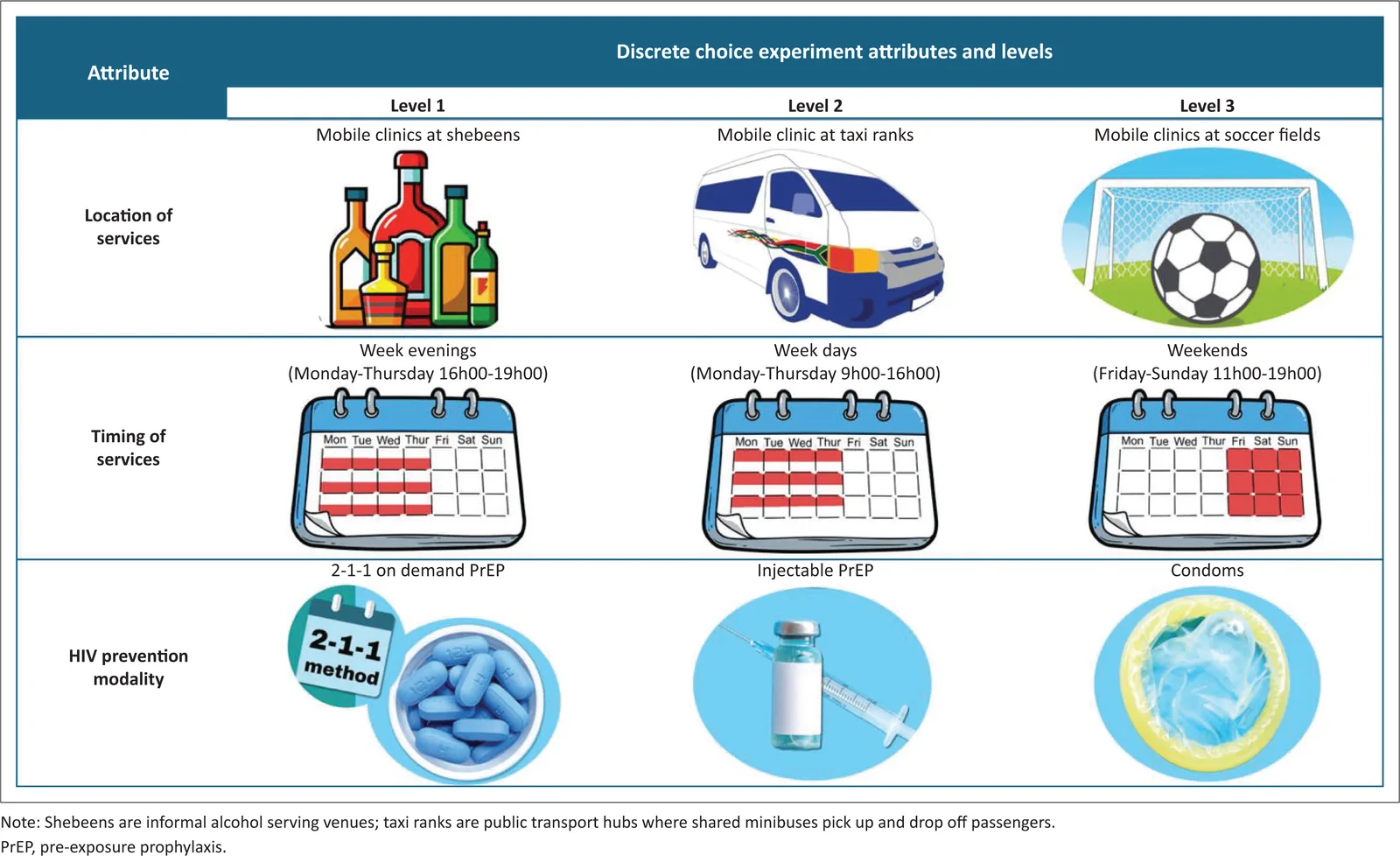
Discrete Choice Experiment attributes and levels, KwaZulu-Natal, South Africa, February 2026.

### Experimental design

The DCE used a full-profile design, with each choice task presenting two hypothetical mobile clinic options defined by all attributes. The experimental design was generated using Sawtooth Software (Lighthouse Studio, Sawtooth Software Inc., Orem, Utah, United States) and employed a D-efficient, non-orthogonal fractional factorial design.^17^ Choice tasks were constructed using the balanced overlap method, which allows some attribute levels to repeat across alternatives within a choice task while maintaining overall level balance and statistical efficiency.^18^ Each participant completed nine choice tasks. A dual-response format was used in which participants first selected their preferred clinic option and then indicated whether they would attend the selected clinic. This allowed opt-out behaviour without requiring an explicit opt-out alternative in the initial choice task.

### Statistical analysis

DCE data were analysed within a random utility framework to estimate preference weights (part-worth utilities) for each attribute level, reflecting their relative contribution to choice. Preferences were estimated using hierarchical Bayesian (HB) multinomial logit models, which allow estimation of individual-level preference weights while accounting for repeated choices within individuals and variation in preferences across respondents.

Attribute levels were effects-coded, such that utilities were normalised to sum to zero within each attribute and interpreted on a relative scale. Part-worth utilities reflect the relative preference for each attribute level within an attribute, with higher values indicating greater preference. Results are presented as mean part-worth utilities across participants, with 95% confidence intervals. Relative attribute importance reflects the contribution of each attribute to participants’ choices relative to the other attributes and is expressed as a percentage.

HB estimation generates individual-level posterior utilities for each respondent, allowing preferences to vary across individuals while being informed by the overall sample distribution. To explore potential patterns in preferences across participant characteristics, mean utilities were summarised descriptively across predefined subgroups based on prior PrEP use (yes/no), alcohol risk category (low–moderate risk, AUDIT-C score 1–3; high risk, AUDIT-C score 4–12), and age group (< 24 years, 25–29 years, ≥ 30 years). These subgroup analyses were exploratory and descriptive; models were not re-estimated within subgroups and no formal interaction tests were conducted. All statistical analyses were conducted in Sawtooth Software, with descriptive statistics summarised using Stata (StataCorp, College Station, Texas, United States). The design, conduct, and reporting of the DCE followed the International Society for Pharmacoeconomics and Outcomes Research Good Research Practices for Conjoint Analysis.^12^

### Ethical considerations

The study was approved by the University of KwaZulu-Natal Biomedical Research Ethics Committee (BREC/00006833/2024) and the Mass General Brigham Institutional Review Board (2024P001106). All participants provided written informed consent prior to participation. During the consent process, participants were informed that the study involved hypothetical service scenarios, that they could decline any question or stop participation at any time, and that discussing HIV prevention, alcohol use, or sexual behavior could cause discomfort. Study data were collected by trained research staff, stored in secure password-protected databases, and de-identified for analysis to protect participant confidentiality.

## Results

Among 240 men eligible for the DCE, 187 (77.9%) were successfully contacted. Of these, 185 completed the survey and were included in the analysis; reasons for non-participation included relocation (*n* = 45, 19%), inability to contact (*n* = 5, 2%), death (*n* = 3, 1%), and refusal (*n* = 2, < 1%). Compared with non-participants, DCE participants were similar in age and prior PrEP use, but were less likely to report hazardous alcohol use and less likely to report high numbers of sexual partners (Online Appendix 1 Table 1-A1).

Participants had a mean age of 27.2 years (standard deviation [s.d.] 4.0). Most were unemployed (70%) and sexually active (97%). Thirty-five per cent reported ever using PrEP, though only 5% were currently taking PrEP at the time of the survey. Alcohol use was common, with 118 participants (64%) categorised as high-risk alcohol use based on an AUDIT-C score ≥ 4.^19^ Behaviours increasing risk for HIV were frequent, including multiple sexual partners and recent condomless sex. Participant characteristics stratified by alcohol risk category are shown in Table 1.

**TABLE 1:** Participant characteristics by alcohol use, KwaZulu-Natal, South Africa, February 2026.

Characteristic	Total (*N* = 185)	%	s.d.	Low/moderate alcohol use (*n* = 67)	%	s.d.	High-risk alcohol use (*n* = 118)	%	s.d.	*P* *
Mean age (years)	27.2	-	4	26.9	-	4	27.3	-	4	0.423
Unemployed	130	70	-	46	67	-	84	71	-	0.718
Sexually active	179	97	-	65	97	-	114	97	-	0.964
Total number of sexual partners in past 12 months										
0–1 partners	48	26	-	23	34	-	25	21	-	0.087
2–5 partners	122	66	-	39	58	-	83	70	-	-
6–12 partners	12	6	-	3	5	-	9	8	-	-
> 12 partners	3	2	-	2	3	-	1	1	-	-
Sex without condom in last 3 months	128	69	-	43	64	-	85	72	-	0.375
Previous diagnosis of STI	67	37	-	24	37	-	43	38	-	0.930
Knowledge of partner’s HIV status	86	46	-	36	54	-	50	42	-	0.199
Testing for HIV within last 12 months	140	76	-	56	84	-	84	71	-	0.060
Ever taken PrEP	65	35	-	20	30	-	45	38	-	0.350
Used PrEP for > 3 months	41	63	-	10	50	-	31	69	-	0.277
Currently taking PrEP	10	5	-	4	6	-	6	5	-	1.000
Methods for preventing HIV										
Cleansing after sex	122	66	-	48	72	-	74	63	-	0.313
Traditional remedies	42	23	-	14	21	-	28	24	-	0.749
Circumcision	119	64	-	43	64	-	76	64	-	0.980
Condoms	142	78	-	55	82	-	87	74	-	0.345
None of the above	7	4	-	1	1	-	6	5	-	0.685

s.d., standard deviation; STI, sexually transmitted infection; PrEP, pre-exposure prophylaxis.

* , *P*-values calculated for comparisons between alcohol consumption groups by Kruskal-Wallis testing.

### Choice task completion and data quality

All enrolled participants completed the full set of nine choice tasks. Median task completion time was 3.9 min (interquartile range [IQR] 3.3–4.7 min). No evidence of widespread dominant response patterns was observed, suggesting that participants engaged meaningfully with the choice tasks.

### Overall preference patterns

HB models were used for primary inference to estimate preferences for mobile HIV prevention service attributes (Figure 2; Online Appendix 1 Table 2-A1). Clinic location was an important driver of participants’ choices. Mobile clinics located at soccer fields were strongly preferred (mean utility 19.4; 95% CI: 13.9 to 24.9), while delivery at shebeens was actively disfavoured (–17.6; 95% CI: –23.2 to –11.9). Taxi ranks were viewed neutrally (–1.8; 95% CI: –6.7 to 3.1). In contrast, the influence of clinic timing was smaller. Participants showed a preference for week evening services (3.5; 95% CI: 1.0 to 6.0), while weekday services were viewed less favourably (–2.6; 95% CI: –5.0 to –0.2) and weekend services were near neutral. Preferences for HIV prevention modality differed substantially across attribute levels. Event-driven (2–1–1) oral PrEP was positively valued (12.3; 95% CI: 1.7 to 22.8), whereas condoms-only delivery was negatively valued (–11.2; 95% CI: –22.1 to –0.2). Injectable PrEP was close to the attribute mean (–1.1; 95% CI: –15.2 to 13.0), suggesting no clear population-level preference and substantial variability in responses.

**FIGURE 2:**
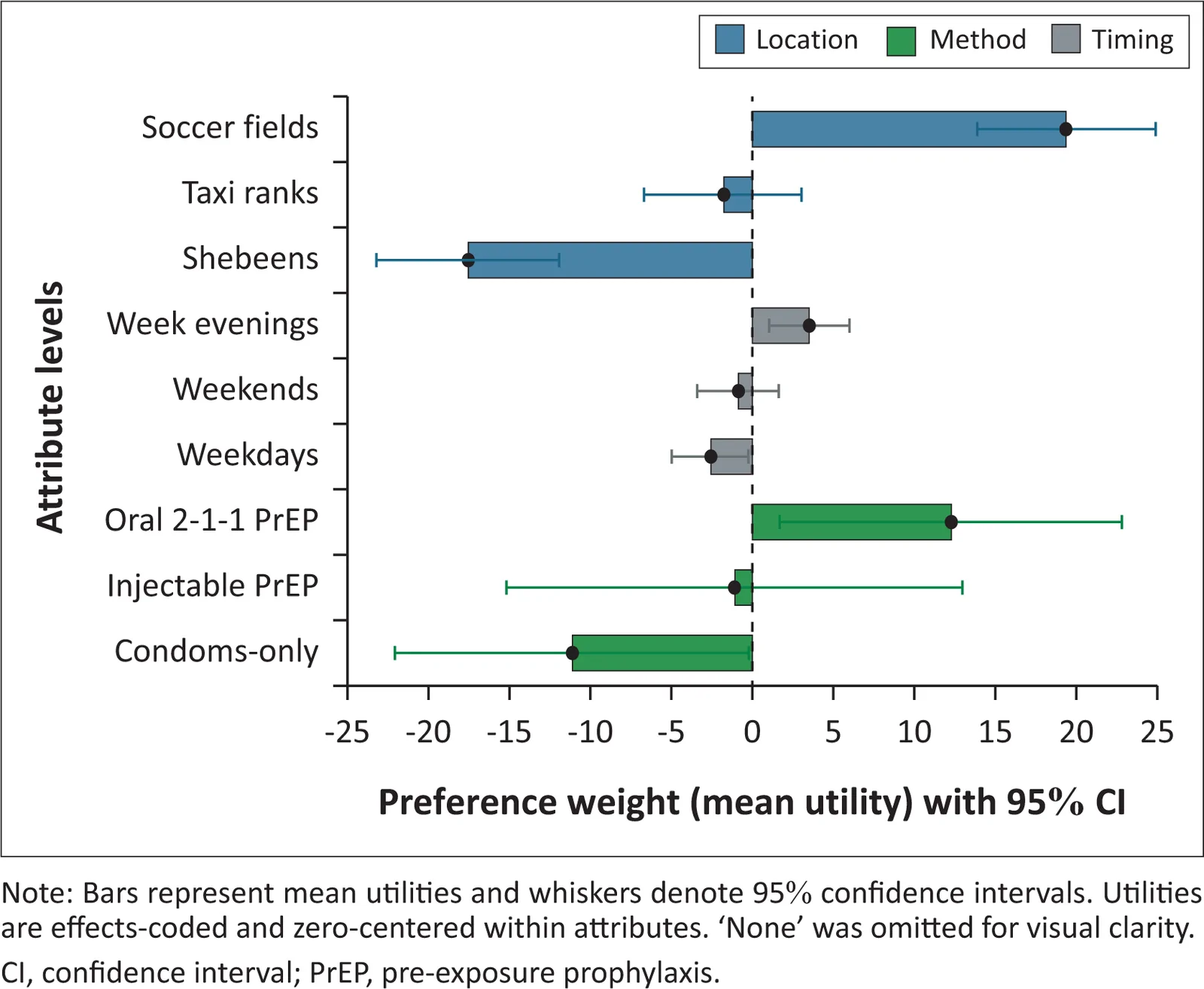
Preference weights from a hierarchical Bayesian model, KwaZulu-Natal, South Africa, February 2026.

Relative attribute importance estimates indicated that HIV prevention modality was the dominant driver of choice (62.8%), followed by clinic location (26.3%) and clinic timing (10.9%) (Figure 3).

**FIGURE 3:**
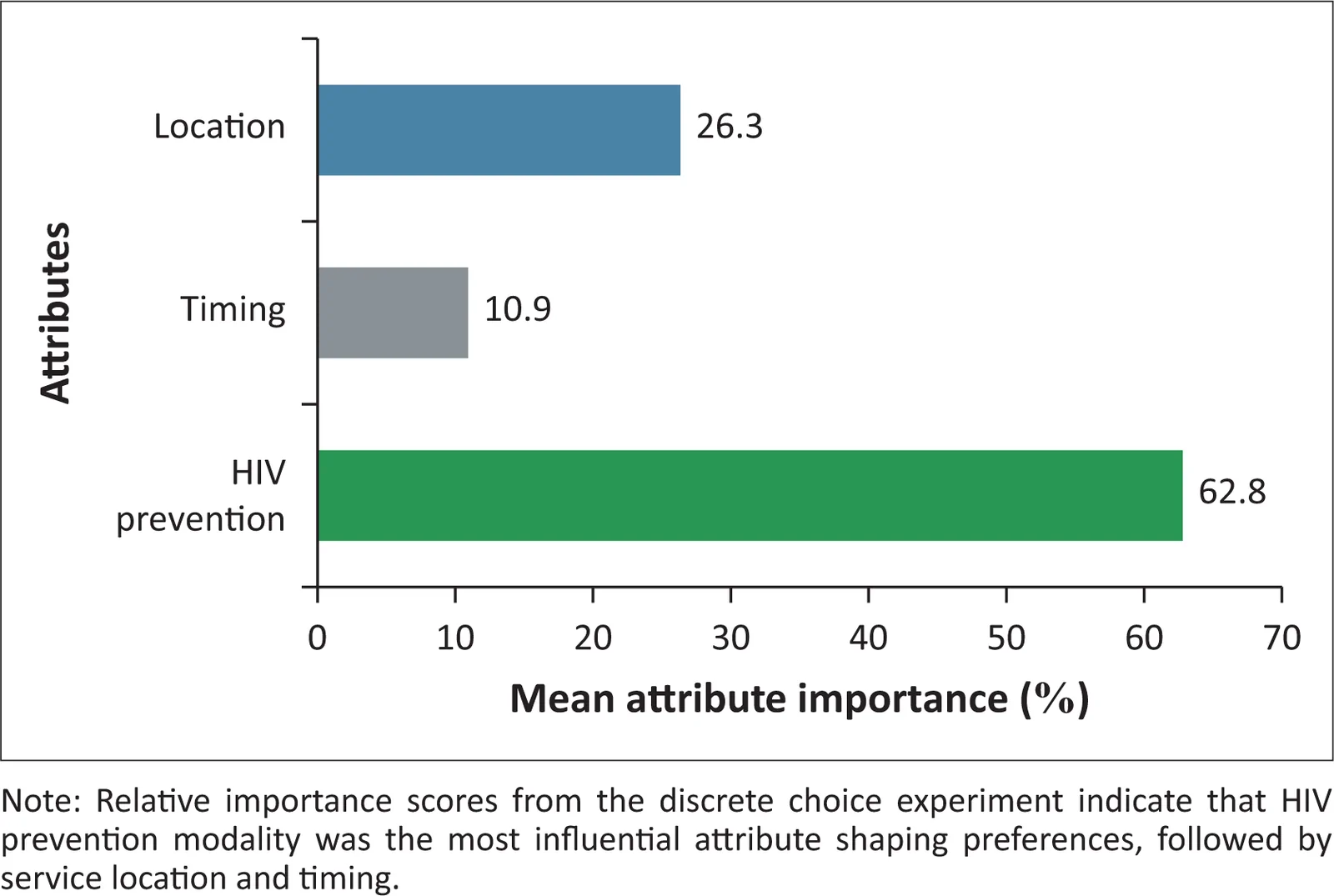
Attribute importance for HIV prevention service preferences (*N* = 185), KwaZulu-Natal, South Africa, February 2026.

### Preference heterogeneity and subgroup patterns

Subgroup summaries of individual-level utilities revealed consistent preferences for clinic location. Across all examined subgroups, including prior PrEP use, alcohol risk and age categories, soccer field-based delivery remained the most highly preferred clinic location (Figure 4).

**FIGURE 4:**
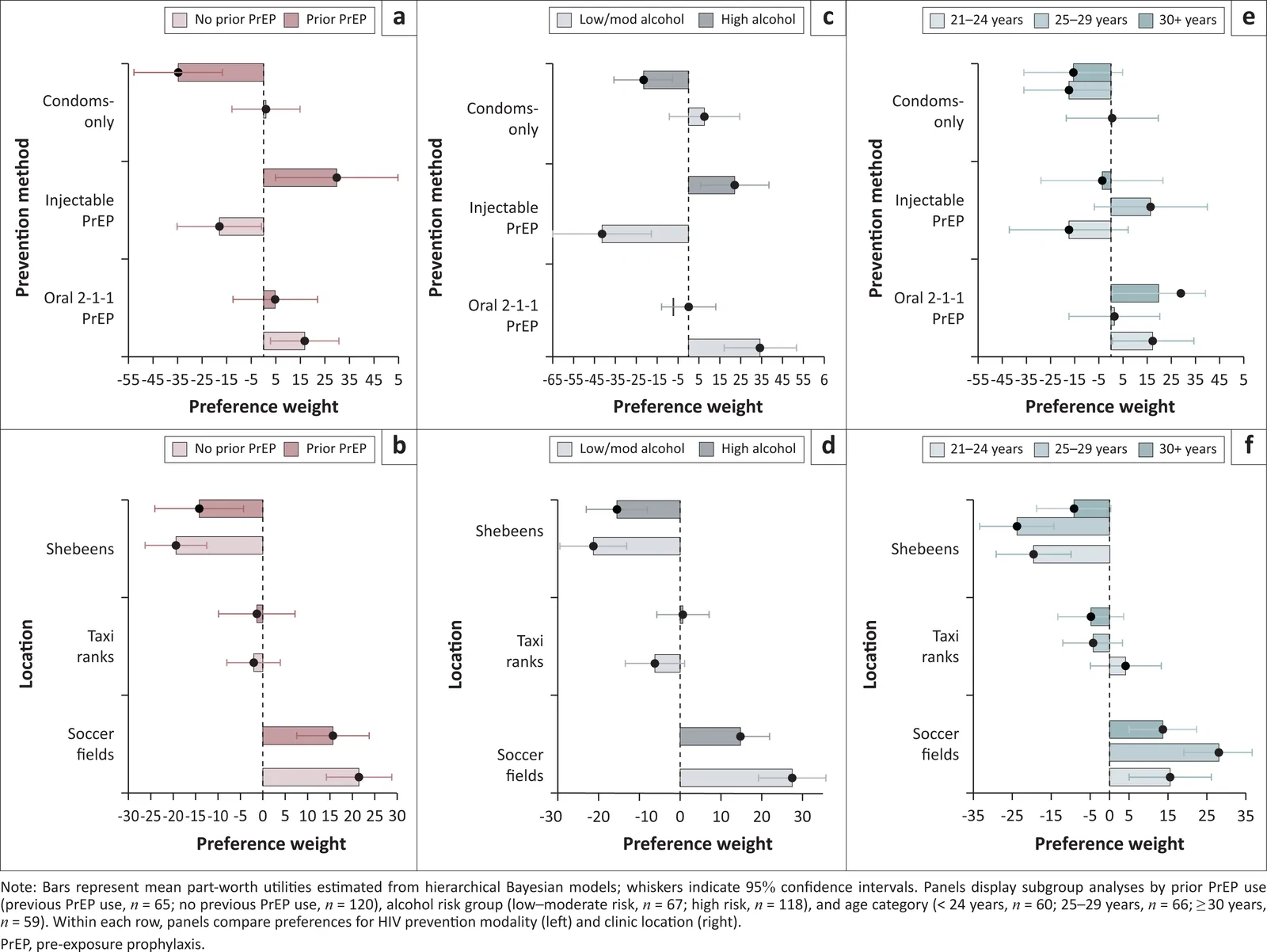
Subgroup preference weights for HIV prevention modalities and clinic location, KwaZulu-Natal, South Africa, February 2026.

By contrast, preferences for HIV prevention modality varied meaningfully across subgroups (Figure 4; Online Appendix 1 Table 3-A1). Participants with prior daily PrEP experience showed a relative preference for injectable PrEP and aversion to condoms-only prevention. In contrast, participants without prior PrEP use preferred event-driven (2–1–1) oral PrEP and were less inclined toward injectable PrEP (Figure 4). Similar patterns were observed by alcohol risk. Participants with high-risk alcohol use showed greater preference for injectable PrEP and aversion to condoms-only prevention. Those with low or moderate alcohol use preferred event-driven oral PrEP and were less inclined toward injectable PrEP (Figure 4). These subgroup characteristics partially overlapped, but represented distinct participant groups. For example, while 69% of participants with prior PrEP use reported high-risk alcohol use, the majority of participants with high-risk alcohol use had not previously used PrEP (Online Appendix 1 Table 4-A1).

Age-based patterns further highlighted heterogeneity in modality preferences. Men younger than 24 years and those older than 30 years showed positive preferences for event-driven oral PrEP, whereas men aged 25–29 years demonstrated a relative preference for injectable PrEP and little preference for event-driven oral PrEP (Figure 4). Condoms-only delivery was negatively valued among participants younger than 30 years but was approximately neutral among participants aged ≥ 30 years. Taken together, these patterns suggest that while preferred service location is largely uniform, preferences for HIV prevention modality differ by prior PrEP exposure, alcohol-related risk, and age.

## Discussion

In this DCE among young men with alcohol use in rural KwaZulu-Natal, HIV prevention modality had the greatest influence on participants’ choices, with clinic location also contributing meaningfully. Clinic timing was considerably less influential. However, preferences for the specific prevention modality were not uniform. Across the full sample, event-driven oral PrEP was positively valued and condoms-only delivery was disfavoured, whereas there was no clear average preference for injectable PrEP because participants’ views varied considerably. Preferences for location were more consistent: soccer field-based mobile clinics were strongly preferred, shebeen-based delivery was clearly disfavoured, and taxi ranks were viewed relatively neutrally. Together, these findings suggest that engaging young men with alcohol use in HIV prevention will require offering choices in a prevention modality within acceptable service locations, whereas expanding operating hours alone is unlikely to meaningfully increase uptake.

The mixed views on prevention modality suggest that a single preferred PrEP option is unlikely to meet the needs of all men. In exploratory subgroup analyses, men with prior PrEP experience and those reporting higher-risk alcohol use tended to prefer injectable PrEP, whereas PrEP-naïve men and those with lower-risk alcohol use tended to prefer event-driven oral PrEP. These findings suggest that modality preferences may be closely tied to prior experience and risk context. Men who had already initiated PrEP appeared more willing to choose injectable formulations, raising the possibility that exposure to oral PrEP may facilitate later uptake of injectables. Conversely, men without prior PrEP experience may prefer starting with event-driven oral PrEP, which may feel less burdensome or more flexible. While long-acting injectable PrEP has been promoted as a strategy to address barriers such as pill fatigue, stigma, and competing priorities,^20^ our results suggest that its appeal is not universal. For PrEP programmes targeting young men with alcohol use, these data indicate that offering both event-driven oral and injectable options may be more effective than prioritising injectables alone, and that early engagement with oral PrEP could serve as an entry point to longer-acting modalities for some men. The modest average utility for injectable PrEP in the overall sample reinforces the importance of framing injectables as one component of differentiated prevention services, with modality choice guided by individual experience, risk context, and preference.

Our data reinforce that men’s engagement with HIV prevention programmes requires availability of socially acceptable services that are compatible with masculine identities.^21,22,23,24^ Previous studies in South Africa have shown that men are more likely to initiate PrEP or test for HIV through community-based services than through traditional clinics, particularly when services are delivered in male-focused or youth-oriented settings.^5,21,25^ However, our findings demonstrate that not all community locations are perceived equally. Despite alcohol-serving venues being epidemiologically high-risk environments and frequented by the study demographic,^26,27^ some men expressed an aversion to receiving HIV prevention services at shebeens. Notably, other studies have demonstrated the feasibility of bar-based PrEP delivery in rural KwaZulu-Natal.^7,28^ Qualitative work from our study population and others has highlighted that shebeens are socially coded as spaces of leisure, masculinity, and peer surveillance, where visible engagement with HIV-related services may carry social costs (Castle et al., manuscript under review). The present findings provide quantitative support for these concerns, indicating that shebeen-based delivery may deter, rather than encourage, young men’s engagement with prevention services. This warrants further investigation.

By contrast, soccer fields appear to function as socially legitimate, identity-congruent spaces in which men are more comfortable accessing health services and information. Sports-based interventions in sub-Saharan Africa have successfully engaged young men in HIV education and testing by embedding health messages within contexts that align with masculine identity, teamwork, and social norms.^29^ Our findings build on this work by demonstrating that, when asked to make explicit trade-offs, men actively prefer soccer fields over other common community venues for receiving HIV-prevention services. Importantly, however, acceptability of venue alone does not guarantee intervention effectiveness. For example, a cluster randomised trial in South Africa found no reduction in HIV incidence or substance use despite delivering prevention interventions through soccer- and vocational-based programmes.^30^ This finding, together with our results, underscores that venue alone may be insufficient and that effective prevention likely requires aligning service location, intervention content, and delivery attributes with men’s preferences.

Clinic timing played a comparatively minor role in decision-making. Preferences for weekday, week evening, or weekend services were modest and centred near zero, suggesting that timing alone may be insufficient to motivate engagement if other aspects of service delivery are misaligned. These findings differ from prior work emphasising extended hours as a key strategy for reaching men, including evidence from the HPTN 071 (PopART) trial showing increased HIV testing uptake during evenings and weekends.^31^ Rather than indicating that timing is unimportant, our results suggest that its influence may be secondary to modality and location when services are delivered in settings men perceive as acceptable. In practice, men may be willing to attend services across a range of times if core aspects of service delivery align with their preferences.

This study has several strengths, including its grounding in formative qualitative research, use of HB methods to capture individual-level preference heterogeneity, and focus on young heterosexual men with alcohol use that remain under-represented in PrEP programmes. By recruiting from a well-characterised longitudinal cohort, we were able to contextualise preferences within documented behavioural and service-use histories. Several limitations should be noted. First, stated preferences in a DCE may not perfectly predict real-world behaviour, particularly as new PrEP modalities become available. Second, participants were drawn from a prior trial population and may have been more familiar with HIV services than men who have never engaged with prevention programmes. Third, the subgroup analyses were exploratory and not designed to formally test interaction effects. Because each subgroup included fewer participants, some estimates were less precise and had wide confidence intervals. These findings should therefore be interpreted as descriptive patterns rather than definitive subgroup differences.

## Conclusion

In conclusion, this DCE suggests that HIV prevention modality is a primary driver of service choice among young men with alcohol use in rural KwaZulu-Natal. While acceptable service locations remain important, delivery in venues that may carry social costs, such as shebeens, was consistently disfavoured. The heterogeneity observed in modality preferences suggests that event-driven oral PrEP may serve as an entry point into biomedical prevention for men who have not previously used PrEP, while long-acting formulations may be more appealing to men already familiar with prevention services. As South Africa expands access to long-acting PrEP formulations, incorporating men’s preferences into service design may help ensure that new biomedical prevention options effectively reach and engage men who have historically been underserved.
